# Amylase production by *Preussia minima*, a fungus of endophytic origin: optimization of fermentation conditions and analysis of fungal secretome by LC-MS

**DOI:** 10.1186/1471-2180-14-55

**Published:** 2014-03-07

**Authors:** Bita Zaferanloo, Shatabdi Bhattacharjee, Mahmood M Ghorbani, Peter J Mahon, Enzo A Palombo

**Affiliations:** 1 Department of Chemistry and Biotechnology, Faculty of Science, Engineering and Technology, Swinburne University of Technology, Hawthorn, VIC 3122, Australia

**Keywords:** *Eremophilia longifolia*, *Preussia*, Endophyte, α- Amylase, Purification, Detergent industry

## Abstract

**Background:**

Environmental screening programs are used to find new enzymes that may be utilized in large-scale industrial processes. Among microbial sources of new enzymes, the rationale for screening fungal endophytes as a potential source of such enzymes relates to the hypothesised mutualistic relationship between the endophyte and its host plant. There is a need for new microbial amylases that are active at low temperature and alkaline conditions as these would find industrial applications as detergents.

**Results:**

An α-amylase produced by *Preussia minima,* isolated from the Australian native plant, *Eremophilia longifolia,* was purified to homogeneity through fractional acetone precipitation and Sephadex G-200 gel filtration, followed by DEAE-Sepharose ion exchange chromatography. The purified α-amylase showed a molecular mass of 70 kDa which was confirmed by zymography. Temperature and pH optima were 25°C and pH 9, respectively. The enzyme was activated and stabilized mainly by the metal ions manganese and calcium. Enzyme activity was also studied using different carbon and nitrogen sources. It was observed that enzyme activity was highest (138 U/mg) with starch as the carbon source and L-asparagine as the nitrogen source. Bioreactor studies showed that enzyme activity was comparable to that obtained in shaker cultures, which encourages scale-up fermentation for enzyme production. Following in-gel digestion of the purified protein by trypsin, a 9-mer peptide was sequenced and analysed by LC-ESI-MS/MS. The partial amino acid sequence of the purified enzyme presented similarity to α-amylase from *Magnaporthe oryzae*.

**Conclusions:**

The findings of the present study indicate that the purified α-amylase exhibits a number of promising properties that make it a strong candidate for application in the detergent industry. To our knowledge, this is the first amylase isolated from a *Preussia minima* strain of endophytic origin.

## Background

Endophytes are microorganisms which live in close association with living plant tissues in a symbiotic relationship. Fungi form a major portion of the endophytic population [1]. Plant endophytic fungi spend the whole or part of their lifecycle colonizing the inside of healthy tissues of the host plants inter-and/or intra-cellularly [2]. This symbiotic relationship protects the host plant from predators whereas the endophytes receive nutrients and living space in return [3]. Endophytic fungi produce several secondary metabolites and enzymes with the potential to hydrolyse several plant-derived macromolecules [4]. Different fungal strains produce different enzymes with natural properties suited to the environmental conditions in which they must act [5]. In many industries, there is a growing demand for new sources of enzymes with various thermostability and pH profiles for different applications [4]. This demand has driven the exploitation of endophytes as enzyme sources for promising industrial applications in agriculture, medicine and food industry [6].

Amylases are ubiquitous enzymes, being widespread in animals, fungi, plants, unicellular eukaryotes and prokaryotes. Amylases account for approximately 30% of world enzyme production [7] and are one of the most important industrial enzymes which are in high demand in various sectors such as food, pharmaceuticals, textiles and detergents. Fungal sources of amylases are mostly terrestrial isolates such as *Aspergillus* species. Their applications include conversion of starch to sugar syrup and the production of cyclo-dextrins in the pharmaceutical industry. The most important amylases for industrial and biotechnological applications are glucoamylases and α-amylases [7]. The latter have the most diverse range of industrial applications that includes brewing, baking, textiles and detergents. Each of these applications requires unique enzyme properties with respect to pH, temperature, specificity and stability [7-9].

α- amylases act as starch-degrading enzymes by catalysing the hydrolysis of internal α-1,4-*O-*glycosidic bonds in polysaccharides with the retention of α-anomeric configuration in the products. They are mostly metalloenymes and require calcium ions for activity, structural integrity and stability. α-amylases can be divided into four basic categories: 1. endoamylases (cleave internal α-1,4 bonds resulting in a α-anomeric products), 2. exoamylases (cleave α- 1,4 or α-1,6 bonds of the external glucose residues resulting in α or β anomeric products, 3. debranching enzymes (hydrolyze α-1,6 bonds exclusively leaving long linear polysaccharides) and 4. transferases (cleave α-1,4 glycosidic bond of the donor molecule and transfer part of the donor to a glycosidic acceptor forming a new glycosidic bond) [7].

In an attempt to identify new amylases with potential application to industry, we have purified and characterised an amylase obtained from a fungus of endophytic origin, *Preussia minima*, isolated from *Eremophila longifolia*. This fungus was previously isolated in our laboratory [4] from plant material supplied by Canopus Corporation (Byrock, New South Wales, Australia) and identified by Dr Bob Chinnock (State Herbarium, Adelaide, South Australia). This native Australian plant was chosen because of its ethno-botanical history and its importance to Australian aboriginal people due to its medicinal properties. Optimization and scale-up studies were carried out to establish the suitability of the fungus for potential industrial scale production of amylase. We further characterized the enzyme using LC-ESI-MS/MS to determine its partial peptide sequence. The characteristics of the enzyme indicated that it has the potential to meet the specifications of several industrial applications, especially the detergent industry.

## Methods

### Preparation of crude extracellular enzyme

The mycelium of *Preussia minima* EL-14, [4], grown on Potato Dextrose Agar (PDA) plates, was inoculated into the first hydrolase-inducing media (which was subsequently shown to induce greater overall protein expression) [10] and incubated at 25°C for 7 days. The media was then centrifuged at 10,000 g for 15 min to separate the mycelia from the broth and the broth filtered through a 0.22 μm filter (Millipore). The sterilized broth was then concentrated by freeze drying and used for characterization of amylase and to study the secretome of *P. minima* EL-14.

A second hydrolase-inducing medium (which was subsequently shown to induce greater amylase production) was used to cultivate growing EL-14 for the purposes of optimization of amylase production. The fungal suspension was prepared with 0.1% Triton X-100 added to 100 ml glucose-aspargine medium [11]. The culture was incubated for 4 days at 25°C with shaking at 220 rpm. To initiate the production of enzyme, 5 ml of the culture grown in the above medium was introduced to 150 ml of inducing media in which glucose was substituted with 4% soluble starch. The mixture was allowed to ferment for 5 days at 28°C. After that, the myecelium were removed by filtration, and the filtrate was saved as the source of crude extracellular amylase.

### Enzyme purification

The culture filtrate was lyophilized and dialyzed against 20 mM Tris–HCl, pH 8. The sample was then precipitated using trichloroacetic acid (TCA)/acetone and applied to a Sephadex G-200 gel filtration column, previously equilibrated and eluted with the same buffer. Fractions were collected and monitored for α-amylase activity. Fractions with activity were then pooled and applied to a DEAE-Sepharose ion exchange column, previously equilibrated with 20 mM Tris–HCl buffer, pH 8, and eluted with a linear concentration gradient (0–17 M) of NaCl in the same buffer. The α-amylase fractions were again pooled and utilized for further characterization, namely SDS-polyacrylamide gel electrophoresis (SDS-PAGE), Bradford assay, zymography and enzyme assay.

### Analysis of extracellular protein content by SDS-PAGE

SDS-PAGE [12] was performed with the Mini-protean® 3 kit (Bio-Rad, USA) using a 12% resolving gel and a 5% stacking gel at 200 V for 45 mins. After electrophoresis, the gel was stained with Coomassie Blue G-250 overnight, de-stained and an image of the gel was captured.

### Zymography

Non-denaturing PAGE (12%) was used to determine homogeneity of the enzyme according to Martinez *et al*. [13]. SDS-PAGE and 2D gel electrophoresis were used to determine the molecular mass of the purified enzyme produced in different conditions under denaturing conditions using 12% acrylamide gel, as described by Laemmli [12]. Staining was carried out with Coomassie Brilliant Blue G-250 and the extracellular protein bands were compared to the Precision PlusProtein Kaleidoscope standards (Bio-Rad) to determine molecular masses. To identify the amylase activity ‘in gel’, 1% (w/v) starch-polymerized non-denaturing PAGE gels were flooded with 0.1 M phosphate-citrate and 0.05 M NaCl buffer (pH 6) and incubated at 39°C for 2 hours. The band with amylase activity was exposed by staining with Lugol’s solution (0.67% KI and 0.33% I_2_).

### Amylase activity and protein content

Amylase activity was determined by measuring the production of reducing sugar from starch using 3, 5-dinitrosalicylic acid (DNS) as described by Miller [14]. The reaction system included 50 μl of enzyme sample and 50 μl of 1.0% starch solution in 0.1 M citrate-phosphate buffer, pH 5.5. After 20 min incubation at 37°C, the reaction was stopped by adding 100 μl of DNS reagent. Blanks contained all the solutions and inactivated (boiled) enzyme sample. One unit of amylase activity was defined as the amount of enzyme that produced 1 μmol of reducing sugar per minute. Protein concentration was measured according to Bradford [15] using bovine serum albumin (BSA) as a standard.

### Effect of different process parameters and additional nutrients on amylase activity

Different conditions, including pH (3, 5, 7, 9 and 10), temperature (9°C, 25°C and 37°C), alternative sources of carbon (maltose, cellulose and glucose) and nitrogen (ammonium nitrate, yeast extract, peptone and tryptophan) in the media and diverse salts (CaCl₂, CoCl₂, MgCl₂, NaCl and MnCl₂) in the phosphate buffer for enzyme assay, were considered to establish the optimum amylase production conditions for EL-14 in the selected hydrolase-inducing medium [11]. The lyophilized ferment broth was used as the crude enzyme solution. After extracellular protein was precipitated using TCA/acetone, the protein pellet was dissolved in 0.1 M of phosphate buffer, then Bradford assays, SDS-PAGE, zymography and enzyme assays were carried out. The same procedures were used for standard fungi *Aspergillus oryzae* (ATCC 10124) and *A. niger* (ATCC 10577) as controls at 25°C and pH 9. Statistical analyses were carried out by analysis of variance (ANOVA) using the Statistical Analysis System (SAS) program. A factorial experiment based on a randomized complete design in three replications was considered for each experiment individually. The Duncan’s Multiple Range Test (DMRT) was used to determine means that differed significantly.

### Bioreactor studies

The α-amylase production by *P. minima* was studied in a 1.4 L bioreactor (Infors AG, Schweiz) with a working volume of 1 L. The bioreactor was equipped with two Rushton-type impellers and baffles and was filled with 1 L of the optimized media. The medium was sterilized at 115°C for 10 min. The final pH of the medium was adjusted by the addition of sterile 1 M HCl or 1 M NaOH. The medium was then inoculated with 10 mL of *P. minima* culture solution. All fermentations were carried out for 5 days at 25°C with the impeller speed adjusted at 180 rpm. Compressed air was sparged into the medium at 1 vvm. Fermentation parameters (temperature, pH and pO_2_) were continuously monitored with microprocessor-controlled probes. Analytical procedures were carried out following scale-up studies. The reducing sugar was estimated by the method of Miller. Protein content was determined by Bradford Assay with BSA as a standard.

### Two-dimensional (2D) gel electrophoresis

Proteins were precipitated in TCA, collected by centrifugation and de-salted using Microcon YM-3 centrifugal filter devices (3,000-nominal-molecular-weight-limit; Millipore, Bedford, MA) according to the protocol described by Peterson et al. [16]. 2D gel electrophoresis was performed using immobilized pH gradient (IPG) strips (11 cm, pH 4 to 7) (GE Healthcare) subjected to isoelectric focusing on an Isoelectric Q focusing machine (Bio-Rad, CA) using the standard protocol recommended by the manufacturer. Gels were fixed in 10% (v/v) methanol, 7% (v/v) acetic acid, stained in Coomassie brilliant blue G-250 and destained in 1% (v/v) acetic acid. The gels were then observed for protein spots.

### Preparation and in-gel digestion of purified amylase fraction/secretome of EL-14

Following filtration of the crude extracellular sample through a 0.22 μm filter, the secretome of *P. minima* was investigated. Thirty three larger protein spots of the EL-14 secretome and purified amylase were excised from replicate 2D electrophoresis and SDS-PAGE gels, respectively, and washed twice with 600 μL of a 1:1 (v/v) de-staining solution (50% of acetonitrile in 50 mM NH_4_HCO_3_) with shaking at 37°C for 30–60 mins. The gel pieces were then dehydrated with 200 μL of 100% acetonitrile and air-dried for 10 min. Proteins in the gel pieces were reduced with 50 μl of freshly prepared 50 mM TCEP, 50 mM NH_4_HCO_3_ for 1 h at 60°C. Reduced proteins were subsequently alkylated by incubation of the gel pieces with freshly prepared 100 mM iodoacetamide in 50 mM NH_4_HCO_3_ for 30 mins at room temperature in the dark. After reduction and alkylation, gel pieces from each protein band were washed twice with 200 μL of a 1:1 (v/v) 100 mM NH_4_HCO_3_/acetonitrile mixture for 15 min, followed by dehydration with 200 μL of 100% acetonitrile, and air-dried for 5 min. Dry gel pieces were rehydrated with 40 μL of 50 mM NH_4_HCO_3_ containing 250 ng of trypsin (Sigma) and then incubated at 37°C overnight [17].

### Peptide sequencing by mass spectrometry (LC-ESI-MS/MS)

Coomassie stained protein bands were excised from the gel and digested with trypsin. The peptides generated from tryptic digestion were analyzed by LC-ESI-MS/MS using an Agilent 1100 Series HPLC coupled to an Agilent LC/MSD Trap XCT Plus Mass Spectrometer fitted with an HPLC Chip cube (Agilent, Palo Alto, CA). Peptides were injected onto a 40 nL Zorbax 300SB-C18 trapping column at a rate of 4 μL min^−1^ and then separated by switching the trap column in-line with the separation column (Zorbax300SB-C18, 75 μm x 43 mm). Samples were separated in 5% solvent B (95% v/v acetonitrile/0.1% v/v formic acid; flow rate 4.00 μL min^−1^). An increase of solvent B from 5% to 15% over 1 min, then a 15 min linear gradient (flow rate 300 nL min^−1^) from 15 to 35% solvent B was performed followed by a step from 35 to 80% solvent B over 0.5 mins and held for 1 min to elute any remaining protein from the column.

The peptide sequences from the MS/MS spectra were identified by the MASCOT searching tool (Matrix Science Ltd., London, UK) against the NCBI nr (National Centre for Biotechnology Information non-redundant) sequence database. The criteria for protein and peptide sequence identification were based on the manufacturer’s definitions (Matrix Science Ltd). Essentially, peptides with a high probability (MASCOT scores exceeding threshold; *p* < 0.05) were referred to as “hits”. Protein scores were derived from peptide ion scores as a non-probabilistic basis for ranking proteins. The primary sequence was analysed using the BLAST database (http://blast.ncbi.nlm.nih.gov/Blast.cgi) against proteins in NCBI nr (all entries).

## Results

### Purification of α-amylase

**Figure 1:**
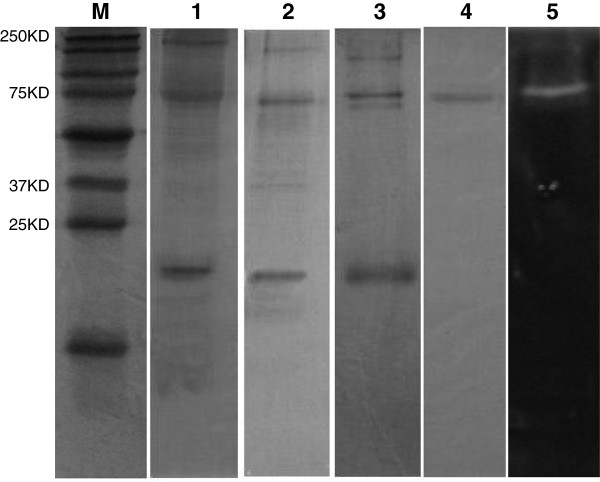
**Electrophoretic analysis of crude and purified samples of EL-14.** Lane **M**: Precision Plus Protein™ Kaleidoscope™ standards (250–10 kDa); Lane **1**: 12% SDS-PAGE of the crude sample; Lane **2**: 12% SDS-PAGE of the sample after TCA concentration; Lane **3**: 12% SDS-PAGE of the sample after gel filtration; Lane **4**: 12% SDS-PAGE of the purified α-amylase after anion exchange; Lane **5**: 12% amylotic zymogram of purified α-amylase developed from protein staining with Lugol’s solution. Zymography was performed to confirm that the purified sample comprised a single band of 70 kDa.

The dialyzed culture filtrate was precipitated and applied to a Sephadex G-200 column. After pooling the activity fractions, the concentrated sample was applied to a DEAE-Sepharose ion exchange column. Data from α-amylase purification are summarized in Table 1. α-amylase purified using TCA/acetone saturation yield up to 5-fold purification. The specific activity at this stage was 235 U/mg with a yield of 80.86%. After Sephadex G-200 filtration, the specific activity of α-amylase increased to 298 U/mg, which further increased to 350U/mg upon DEAE-Sepharose ion exchange chromatography. Overall, the enzyme was purified approximately 7-fold, with 55.11% recovery. The total protein and enzyme activity was measured after each step of purification and a single band was observed in SDS-PAGE and zymography (Figure 1).

**Table 1:** **Purification summary for extracellular α-amylase produced by *Preussia minima***

**Purification step**	**Total activity (U)**	**Total protein (mg)**	**Specific activity (U/mg)**	**Purification (fold)**	**Yield (%)**
Crude extract	1270	27.02	47	1	100
TCA concentration	1027	4.37	235	5	80.86
Gel filtration	834.4	2.8	298	6.34	65.7
Anion exchange	700	0.9	350	7.44	55.11

### Effect of temperature on α-amylase activity

**Figure 2:**
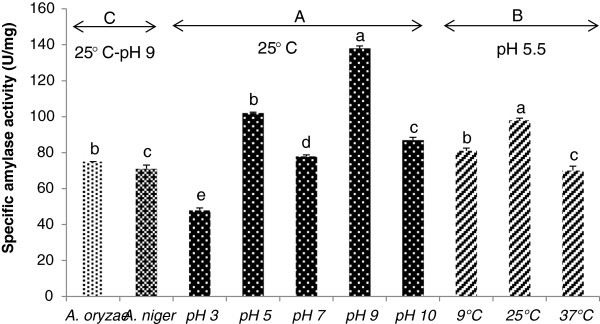
**Effect of pH and temperature on specific amylase activity of EL-14 and standard. (A)** EL-14 was grown at different pH and specific amylase activity was determined. **(B)** EL-14 was grown at different temperatures and specific amylase activity was determined. **(C)** standards (*A. oryzae* and *A. niger*) were grown at the same conditions to compare their activity with *P. minima*. The crude extracellular sample (EL-14) exhibited the highest activity at 25°C and pH 9, significantly more than standard. Means followed by the same letter within a column are not significantly different at *P* < 0.01 according to the Duncan multiple range test.

The optimum temperature of the α-amylase was evaluated by measuring the specific activity of extracellular extracts at different temperatures at pH 5.5. The enzyme presented a temperature optimum at 25°C. The optimum value for α-amylase production was observed at 98 U/mg (Figure 2). Enzyme activity at low temperature was notable as well.

### Effect of pH on α-amylase activity

**Figure 3:**
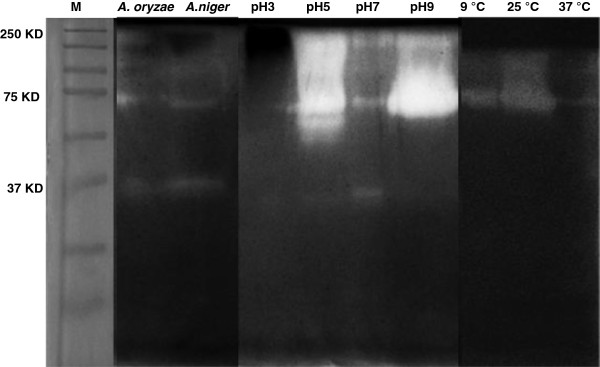
**Qualitative assessment of amylase activity by zymography.** Zymography was performed to confirm that α-amylase activity at pH 9 and 25°C was optimal, in comparison with standards (*A. oryzae and A. niger*). M = molecular weight markers. A common band was detected at around 70 kDa for *P. minima*.

The effect of pH was also investigated. The enzyme was pre-incubated at optimum temperature 25°C and different pH. The pH optimum was 9.0 with a specific activity of 138 U/mg (Figure 2). α-amylases from most fungi are known to have pH optima in the acid to neutral range. They are generally stable in a wide range of pH from 4–11 [18]. The particular enzyme investigated in this study showed optimum activity in alkaline conditions (similar to bacteria) and high activity at lower temperature (similar to other fungi). Thus, the combination of optimum temperature and pH make this fungal α-amylase unique. All the results were confirmed by zymography (Figure 3). The significance of each factor was determined by *F* values and *P* values (Table 2). The model *P* value <0.0001 implied the model was significant and all the factors and their interactions with *P* values less than 0.01 were significant (α = 0.01). Of particular interest was that the enzyme activity of *P. minima* was greater than that of the standard fungi, *A. oryzae* and *A. niger*, grown under the same conditions (Figure 2).

**Table 2:** Test of significant effects of three factors (EL-14 growing in different conditions, temperature and pH) on amylase activity

**Source**	***df***	**Sum of squares**	**Mean square**	***F *** **value**	***P *** **value**^1^
Model	127	103971.393	818.672	3344.364	<0.0001
pH	3	28635.049	9545.016	38992.408	<0.0001
temp	3	7229.945	2409.982	9845.032	<0.0001
sample	7	24030.581	3432.940	14023.926	<0.0001
pH*temp	9	8092.023	899.114	3672.975	<0.0001
pH*sample	21	5643.513	268.739	1097.826	<0.0001
temp*sample	21	11290.784	537.656	2196.383	<0.0001
pH*temp*sample	63	19049.497	302.373	1235.226	<0.0001
Error	256	62.667	.245		
Corrected Total	383	104034.060			

^1^*P* values less than 0.01 are significant.

Probability P (>F) <0.0001; R-Square = 0.999.

### Effect of different metal ions on amylase activity

**Figure 4:**
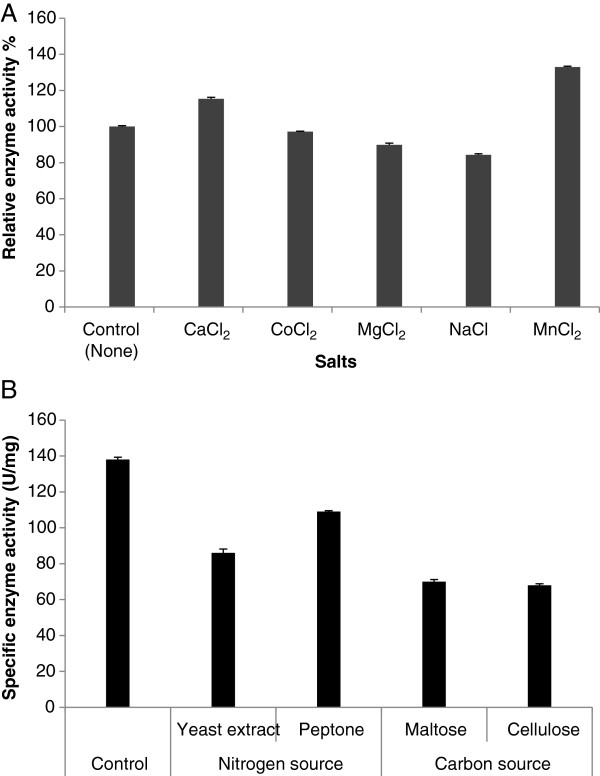
**Effect of metal ions and different carbon and nitrogen sources on amylase activity. (A)** The relative enzyme activities were measured at optimum of pH and temperature and enzyme activity without metal ions was taken as 100%. **(B)** Effects of different carbon and nitrogen sources at optimum conditions (25°C and pH 9) on α-amylase production of *P. minima*. The hydrolase-inducing medium of Nyugen et al. [11] was used as a control. Where nitrogen sources were replaced, starch was the carbon source (as per the control medium); where carbon sources were replaced, L-asparagine was the nitrogen source (as per the control medium). Means followed by the same letter within a column are not significantly different at *P* < 0.01 according to the Duncan multiple range test.

Enzyme assays were performed under two different conditions, the normal enzyme assay described previously and assays performed with addition of different metal ions (Na^+^, Mg^2+^, Ca^2+^, Co^2+^, Mn^2+^) at 2.5 mM. The chloride salts of these metal ions were used (CaCl₂, CoCl₂, MgCl₂, NaCl and MnCl₂). The amylase activity was measured at optimum of pH and temperature in the presence of these metal ions. The relative activity of the enzyme was compared with the activity obtained using 0.1 M citrate-phosphate buffer. Results demonstrated that CaCl₂ and MnCl₂ increased the total amylase activity whereas MgCl₂ and NaCl inhibited the enzyme activity in comparison to the control (Figure 4A).

### Effect of using different sources of carbon and nitrogen on amylase activity

The effects of different nitrogen and carbon sources were studied at optimum conditions [11] and it was observed that inclusion of starch and L-asparagine (control media) produced higher specific activity (138 U/mg). Replacement with three different carbon (maltose, cellulose, glucose) and nitrogen sources (ammonium nitrate, yeast extract, peptone and tryptophan) did not increase production of amylase. Results based on measuring the specific enzyme activity are summarised in Figure 4B. Zymography was carried out for all samples and results confirmed that highest activity was observed when using starch and L-asparagine as carbon and nitrogen sources, respectively (data not shown). Statistical analysis showed that three factors including media (different sources of carbon and nitrogen), pH and temperature and their interactions had significant effects on amylase activity (data not shown). The average of activities using different sources of carbon and nitrogen were compared by the Duncan multiple range test and showed that starch as the carbon source and L-asparagine as nitrogen source produced the greatest amylase activity at 25°C and pH 9.

### Production of α-amylase in 1.4 L bioreactor

The α-amylase production was studied under controlled temperature and pH conditions (optimal conditions for *P. minima*) of 25°C and pH 5 [11] in a 1.4-L bioreactor with 1 L of medium for 5 days. These reaction conditions were found best for growth of the fungus. The total enzyme activity obtained using the bioreactor was 212 U/ml, which was comparable to the enzyme activity in shaker flasks (214 U/ml). Therefore, it can be concluded that laboratory scale-up of *P. minima* does not affect the α-amylase activity.

### Mass spectrometry and peptide sequencing

**Figure 5:**
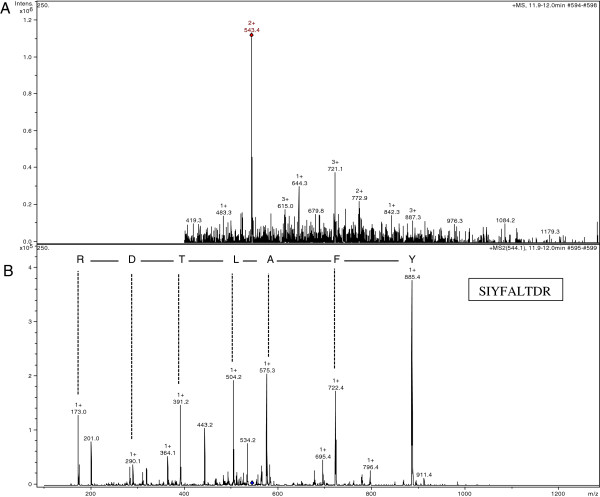
**MS and MS/MS spectrum on LC-ESI-MS/MS. (A)** Tryptic digest peptide spectrum of purified 70 kDa band from *P. minima.***(B)** MS/MS spectrum of precursor peak (543.4). The data are representative of three independent experiments.

A peptide spectrum obtained from in-gel tryptic digestion of purified α-amylase gave 13 prominent ions (Figure 5A). These peak list generated from the spectrum was used for protein identification against the NCBI nr database using the MASCOT search engine, resulting in one hit. The signal 543.4 was chosen as the target to analyse in the MS mode to identify the precursor ion formed by the ESI ion source. The fragments and fingerprint information of the MS/MS spectrum of the precursor peak were analysed and the results with scores higher than 54 identified the amino acid sequence of a 9-mer peptide as SIYFALTDR (Figure 5B). The peptide sequence was used to search the NCBI database which identified similarity to α-amylase [NCBI nr: gi 389646691] from *Magnaporthe oryzae*. Given that this sequence is a provisional RefSeq in the NBCI database and has not been subject to final review, we performed an additional search of the UniProt database using the Protein Pilot search engine which yielded the same match.

### Protein identification and secretome study of *Preussia minima*

**Figure 6:**
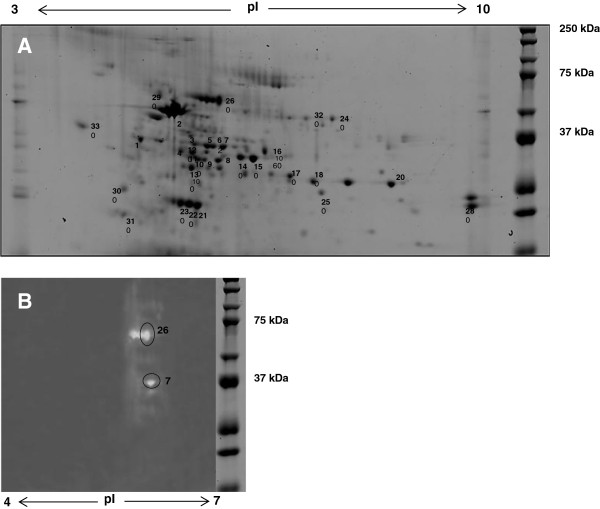
**Two-dimensional gel electropherogram and its amylase zymogram. (A)** 2D gel was scanned with a Typhoon FLA 9000 laser scanner (GE Healthcare) using a no emissions filter, PMT 600, Laser Red (633) and normal sensitivity. Crude extracellular proteins (approx. 200 μg) from the supernatant of *P. minima* following growth in the first hydrolase-inducing medium for 7 days were used to perform 2D gel electrophoresis. Proteins were visualized on a 12% SDS-PAGE gel stained with Coomassie Blue and identified by LC-ESI-MS/MS as indicated in Table 3. **(B)** Amylase zymogram (pI 4 to 7) produced after 2D gel electrophoresis of protein using crude sample*.* No amylase activity was detected above around pI 6. Protein in spots 26 and 7 could be assigned to α-amylase in the NCBI nr database by LC-MS/MS analysis (Table 3). Two spots beside spot 26 could be isomers of amylase with different pI.

Separating proteins in the *P. minima* supernatant by their molecular weight as well as the isoelectric point was an effective way of isolating proteins with amylase activity from other abundant proteins occurring at the same molecular weight. Unambiguous identification of purified protein with amylase activity (Figure 6B, spot 26) was performed using ESI-LC MS/MS. The other 32 protein spots in the 2D gel (Figure 6A) which were identified are listed in Table 3.

**Table 3:** **Proteins in the secretome of *Preussia minima *** **identified by LC MS/MS and compared to proteins in the NCBI database**^**1 **^**by BLAST searching**

**Spot No**	**Enzyme category, protein name/function**^**2**^	**Target substrate**	**Species**	**Accession No.**	**Mascot score**
1	Xylanse	Xylan	*Bacillus subtilis*	gi 139865	65
2	Chitinase	Chitin	*Bacillus subtilis*	gi 16079742	59
3	Pectate lyase	Pectin	*Bacillus subtilis*	gi 16080548	72
4	Phosphoribosyl amino imidazole Carboxylase catalytic subunit		*Legionella lonfbeachae*	gi 270158584	76
5	Natto kinase		*Bacillus subtilis*	gi 14422313	66
6	Protease	Protein	*Xenopus tropicalis*	gi 58332100	51
7	Alpha amylase	Starch	*Bacillus subtilis*	gi 255767082	85
8	Cellulase	Cellulose	*Bacillus subtilis*	gi 39777	67
9	Nicotinate-Nucleotide pyrophosphorylase		*Bacillus subtilis*	gi 16079838	77
10	Endo 1,4- beta mannosidase	Mannan	*Bacillus amyloliquefaciens*	gi 384158076	52
11	Beta- 1,4-mannase	Mannan	*Bacillus pumilus*	gi 347311566	82
12	Endo-beta 1,4 mannase	Mannan	*Bacillus subtilis*	gi 84688836	396
13	Mannase	Mannan	*Bacillus licheniformis*	gi 239634423	73
14	Hypothetical protein		*Zymoseptoria tritici*	gi 398404980	54
15	Bacillo peptidase	Protein	*Bacillus subtilis*	gi 62946514	125
16	Hypothetical protein		*Colletotrichum*	gi 429850915	67
17	Arginase	Xylan	*Bacillus subtilis*	gi 16081084	87
18	Arabian-endo1,5-alpha-L-arabinase	Xylan	*Bacillus subtilis*	gi 1770013	118
19	Neutral protease	Protein	*Bacillus mesenterious*	gi 56405351	69
20	Bacillo peptidase F	Protein	*B. mesenterious*	gi 142609	110
21	Unnamed protein product		*Cyanophora paradoxa*	gi 11416	69
22	Endo-1,4-beta-glucanase	Xyloglucan	*Bacillus subtilis*	gi 16079934	80
23	Catalase	Hydrogen peroxide	*Aspergillus fumigatus*	gi 1857716	216
24	VBS		*Aspergillus flavus*	gi 46370484	70
25	Aldose 1- epimerase	Glucose	*Pyrenophora tritici*	gi 189190	81
**26**	**Alpha amylase**	Starch	*Magnaporthe oryzae*	gi 389646691	56
27	Hypothetical protein		*Gibberella zea*	gi 46126833	84
28	Extracellular neutral metalloprotease	Protein	*Bacillus subtilis*	gi 160789534	77
29	Catalase	Hydrogen peroxide	*Glomerella graminicola*	gi 310791536	107
30	Cellobiose dehydrogenase	Cellulose	*Cochliobolus sativus*	gi 451856	92
31	Hypothetical protein		*Aspergillus terreus*	gi 115388337	78
32	Mycelial catalase	Hydrogen peroxide	*Neosartorya fischeri*	gi 119474019	105
33	Hypothetical protein SMAC_03015		*Sordaria macrospora*	gi|336269335	68

^1^Assignment of proteins to all the entries in the NCBI database (http://www.ncbi.nlm.nih.gov/) was determined by ESI-LC-MS/MS of protein spots or bands of 1D and 2D gels. Identification.

^2^Names or predicted functions of conserved domains in proteins in the NCBI nr database (http://www.ncbi.nlm.nih.gov/) to which significant peptide matches (*P <* 0.05) were made using the Mascot search engine. The enzyme in bold (spot 26) denotes the 70 kDa α-amylase described above.

## Discussion

The present study was undertaken to investigate a newly isolated α-amylase from a fungus of endophytic origin, *Preussia minima*, and the potential opportunities it may offer as an additive, particularly for the detergent industry. The purification of α-amylase was performed from crude extracts of culture and approximately 7-fold greater enzyme concentration than the crude enzyme with 55.11% recovery was achieved. Since specific activity is affected by the different conditions used in fermentation and purification steps, various values of activity were reported [19,20]. The lower purification yield of α-amylase could be attributed to higher loss of enzyme during downstream processing or, alternatively, lower initial protein concentration of the crude extract used for the purification process [20]. The activity of α-amylase can be increased using various activators [19].

Enzyme assays were performed of *P. minima* culture supernatants following incubation in the second hydrolase-inducing medium [11] at different temperatures and pH. The optimum temperature and pH based on specific enzyme activity were 25°C and 9, respectively; however, activity at low temperature was also notable. The optimum temperature and pH conditions of amylase production are likely to reflect the climatic conditions found in environments inhabited by the original host plant [4]. The name *Eremophila* means ‘desert-loving’ and this genus is usually found throughout Australia, predominantly in arid conditions.

Specific activity (138 U/ mg) at pH 9 was remarkable in comparison with activities previously reported [8,9,18,21-24]. These results were confirmed by zymography. α-amylases from most fungi exhibits pH optima in the acid to neutral range; however, this enzyme appears to be exceptional with a pH optimum of 9.0. Though slightly alkaline, α-amylases have been reported to be stable in a wide pH range [18]. Amylase activity under these conditions of low optimum temperature and alkaline pH optimum would be desirable for the application of this enzyme in the detergent industry as an additive to remove starch-based stains [25].

The effect of metal ions on total amylase activity was studied. Results demonstrated that CaCl₂ and MnCl₂ increased the total amylase activity in comparison to the control, confirming previous reports which indicated that amylases are mostly metalloenymes and require calcium and manganese ions for activity, structural integrity and stability [7,9,18]. Calcium enhances amylase activity by its interaction with negatively charged amino acid residues such as aspartic and glutamic acids [22]. Magnesium and sodium ions were found to inhibit amylase activity and similar observations were made by Varalakshmi *et al*. [9] and Reyed [26]. The results obtained with MnCl₂ suggest that this salt can be a strong candidate as a culture additive to increase enzyme production. The effects of different nitrogen and carbon sources suggested that inclusion of starch and L-asparagine (in the standard medium) produced higher specific activity (138 U/mg). Replacement with three different carbon (maltose, cellulose and glucose) and nitrogen sources (ammonium nitrate, yeast extract, peptone and tryptophan) did not increase production of amylase. These results were confirmed by zymography. We observed that fungal growth was superior when the medium was supplemented with specific carbon and nitrogen sources, which reflected the reported levels of enzyme production. This suggests that enzyme activity is linked to biomass production. Many previous reports showed the same effect on enzyme activity by changing the conditions of fermentation [8,9,21,24]. Since fungi do not naturally produce enzymes at levels high enough for commercial purposes, fermentation is undertaken to increase the secretion of target enzymes to levels that are economically sustainable. Consequently, environmental screening programs are used to seek enzymes from various environments, with the view to express these enzymes in highly secreting production hosts [27]. In the present study, different sources of carbon and nitrogen showed significant effects in both amylase production and enzyme activity.

In the bioreactor studies, total enzyme activity (212 U/ml) was comparable to that in shaker cultures (214 U/ml). Thus, from the current studies in the bioreactor, it was concluded that the process for the production of α-amylase from *P. minima* can be optimized successfully in a bioreactor with no loss in enzyme activity, which has significant implications for practical applications in industry.

The secretome of *P. minima* was analysed using gel electrophoresis and mass spectrometry. As the genome sequence was unavailable, cross-species identification and zymography assisted the analysis. Most of the identified proteins within the *P. minima* secretome are enzymes involved in the degradation of plant cell wall polymers (starch, cellulose, lignin, pectin and proteins). A diverse range of other enzymes, as well as some proteins with unknown functions, were also identified from the secretome study. Two of the protein spots on the 2D gel were identified as α-amylase. However, a few spots could be seen clustered around the same molecular weight of one of the spots (spot 26) but with slightly different pI values, which could possibly be isoenzymes of α-amylase. Amylase activity was shown by sharp common bands on the 1D amylase zymogram (approximately 70 kDa) at the approximate molecular weight of the identified amylase spot 26 on the 2D gel. The prominent spot was identified as α-amylase based on LC-ESI-MS/MS data from a protein spot at approximately 70 kDa, pI 6 on the 2D gel.

To aid metabolism and subsequent survival under different environmental conditions, endophytic fungi secrete several polymer-degrading enzymes, many of which were identified in the *P. minima* secretome. One such polymer is xylan, which is abundant in the leaves of dicotyledons such as *Eremopholia* species. This requires the action of numerous enzymes such as endo- 1, 4- β-xylanases, for its complete degradation. It acts by cleaving the xylose sugar backbone. Xylanase was also found in the secretome of other fungi, including *Podospora anserine* and *Doratomyces stemonitis*[16]. Cellulose forms an integral part of plant cell wall where it is covalently linked with lignin. The presence of cellulase in the secretome of *P. minima* implies the need of the enzyme to break down plant material by the fungus to obtain nutrients. Another enzyme identified in the *P. minima* secretome was pectinase, which is involved in the degradation of pectin, an indigestible polysaccharide in leaves. The presence of pectin-degrading enzymes in the secretome of *P. minima* could be reflective of high levels of pectin contained within *E. longifolia* leaves. In previous studies, pectinase was identified in other industrial fungi such as *Aspergillus* sp. and *D. stemonitis*[16]. Mannase was also found in the secretome of *P. minima*. Mannan forms a component of the cell wall matrix of dicotyledonous leaves and the mannase acts by cleaving within the mannose backbone of the mannan polymer. Two different kinds of proteases were found in the secretome of *P. minima* that could allow the fungus to increase the efficiency of the degradation of the plant cell wall matrix. Metalloproteases are endoproteases that cleave within amino acid chains and enable fungi to utilize proteins during the digestion process. Proteases have been found in the secretomes of other filamentous fungi, including *Aspergillus oryzae*[28], *Aspergillus niger*[29], *Botrytis cinerea*[30] and *Trichoderma reesei*[31].

As the genome of *P. minima* has not been sequenced, all protein assignments in *P. minima* secretome were made by cross-species identification based on sequence similarities to proteins from other fungal and bacterial species in the NCBI database. The greatest number of assignments (nine) was to proteins from *Bacillus subtilis.* Many of the other proteins were from fungi *Magnaporthe oryzae* and *Aspergillus* sp. Over twenty different types of enzymes have been identified in the secretome of *P. minima* as a result of our work, though α-amylase dominated the secretome. The other proteins were mainly enzymes that break down cellulose, lignin, pectin and protein which is reflective of the fungal endophytic lifestyle. However, there were many small protein spots that were left unidentified as good quality MS/MS spectra could not be assigned confidently to any known protein in the NCBI database. These unidentified proteins might be enzymes that have complementary activity to the enzymes already identified in the secretome, thereby, increasing their access to their target substrates.

## Conclusions

The present study described, for the first time, the purification and characterization of an α-amylase from a strain of *Preussia minima* isolated from the Australian native plant, *Eremophila longifolia*. The techniques of gel electrophoresis, zymography and mass spectrometry allowed us to characterize α-amylase and identify other proteins in the secretome of this fungus. The results obtained here show that the main extracellular enzyme secreted by *P. minima* is an α-amylase. The yield and activity of this enzyme was not only enhanced by the nature of carbon and nitrogen sources but also by specific pH of the fermentation medium and incubation temperature. The alkaline pH optima make it suitable for industrial production. The successful scale-up study encourages its effective utilization for large-scale industrial processes. Amylases are highly sought after in the food industry for the production of various syrups and in the detergent industry as an additive to remove starch based dirt. Therefore, the α-amylase presented in this work may be considered as a potential strong candidate for future application in the detergent industry where alkaline amylases are particularly in demand, as most of the known and commonly used industrial fungal amylases are active in acidic conditions [21]. Further characterisation of this enzyme will include assessing its activity and performance in the presence of surfactants to determine its suitability for use in the detergent industry. Additionally, detailed comparison of the *P. minima* secretome with other reported endophytic fungal secretomes may yield valuable insights into the host plant-fungal association. As the first proteomic study of the secretome of *P. minima*, the array of enzymes identified could have the potential to increase the efficiency of various industrial processes in the future.

## Competing interests

The authors declare that they have no competing interests.

## Authors’ contributions

BZ, SB, PM and EP designed the experiments, evaluated the results and wrote and corrected the manuscript. BZ, SB and MG performed the experiments and evaluated the results. All authors read and approved the final manuscript.
